# Single-cell RNA-seq analysis decodes the kidney microenvironment induced by polystyrene microplastics in mice receiving a high-fat diet

**DOI:** 10.1186/s12951-023-02266-7

**Published:** 2024-01-03

**Authors:** Wenhao Xu, Shiqi Ye, Wangrui Liu, Huaqi Guo, Linhui Zhang, Shiyin Wei, Aihetaimujiang Anwaier, Kun Chang, Guilherme Malafaia, Hailiang Zhang, Dingwei Ye, Gang Wei

**Affiliations:** 1 Department of Urology, Fudan University Shanghai Cancer Center, Shanghai, 200032 People’s Republic of China; 2 Department of Oncology, Shanghai Medical College, Fudan University, Shanghai, 200032 People’s Republic of China; 3 Shanghai Genitourinary Cancer Institute, Shanghai, 200032 People’s Republic of China; 4 Department of Interventional Oncology, Renji Hospital, Shanghai Jiao Tong University School of Medicine, Shanghai, 200127 People’s Republic of China; 5 Department of Pulmonary and Critical Care Medicine, The Ninth People’s Hospital of Shanghai Jiao Tong University School of Medicine, Shanghai, 200011 China; 6 Affiliated Hospital of Youjiang Medical University for Nationalities, Baise, 533000 China; 7 Laboratory of Toxicology Applied to the Environment, Goiano Federal Institute - Urutaí Campus, Rodovia Geraldo Silva Nascimento, 2,5 Km, Zona Rural, Urutaí, GO Brazil; 8 Beijing Key Laboratory of Diabetes Research and Care, Department of Endocrinology, Beijing Tongren Hospital, Beijing Diabetes Institute, Capital Medical University, Beijing, 100730 China

**Keywords:** Kidney microenvironment, Polystyrene microplastics (PS-MPs), Single-cell RNA sequencing (scRNA-seq), High-fat diet (HFD), Renal fibrosis, PF4^+^ macrophages

## Abstract

In recent years, the environmental health issue of microplastics has aroused an increasingly significant concern. Some studies suggested that exposure to polystyrene microplastics (PS-MPs) may lead to renal inflammation and oxidative stress in animals. However, little is known about the essential effects of PS-MPs with high-fat diet (HFD) on renal development and microenvironment. In this study, we provided the single-cell transcriptomic landscape of the kidney microenvironment induced by PS-MPs and HFD in mouse models by unbiased single-cell RNA sequencing (scRNA-seq). The kidney injury cell atlases in mice were evaluated after continued PS-MPs exposure, or HFD treated for 35 days. Results showed that PS-MPs plus HFD treatment aggravated the kidney injury and profibrotic microenvironment, reshaping mouse kidney cellular components. First, we found that PS-MPs plus HFD treatment acted on extracellular matrix organization of renal epithelial cells, specifically the proximal and distal convoluted tubule cells, to inhibit renal development and induce ROS-driven carcinogenesis. Second, PS-MPs plus HFD treatment induced activated PI3K-Akt, MAPK, and IL-17 signaling pathways in endothelial cells. Besides, PS-MPs plus HFD treatment markedly increased the proportions of CD8^+^ effector T cells and proliferating T cells. Notably, mononuclear phagocytes exhibited substantial remodeling and enriched in oxidative phosphorylation and chemical carcinogenesis pathways after PS-MPs plus HFD treatment, typified by alterations tissue-resident M2-like PF4^+^ macrophages. Multispectral immunofluorescence and immunohistochemistry identified PF4^+^ macrophages in clear cell renal cell carcinoma (ccRCC) and adjacent normal tissues, indicating that activate PF4^+^ macrophages might regulate the profibrotic and pro-tumorigenic microenvironment after renal injury. In conclusion, this study first systematically revealed molecular variation of renal cells and immune cells in mice kidney microenvironment induced by PS-MPs and HFD with the scRNA-seq approach, which provided a molecular basis for decoding the effects of PS-MPs on genitourinary injury and understanding their potential profibrotic and carcinogenesis in mammals.

**Graphical Abstract:**

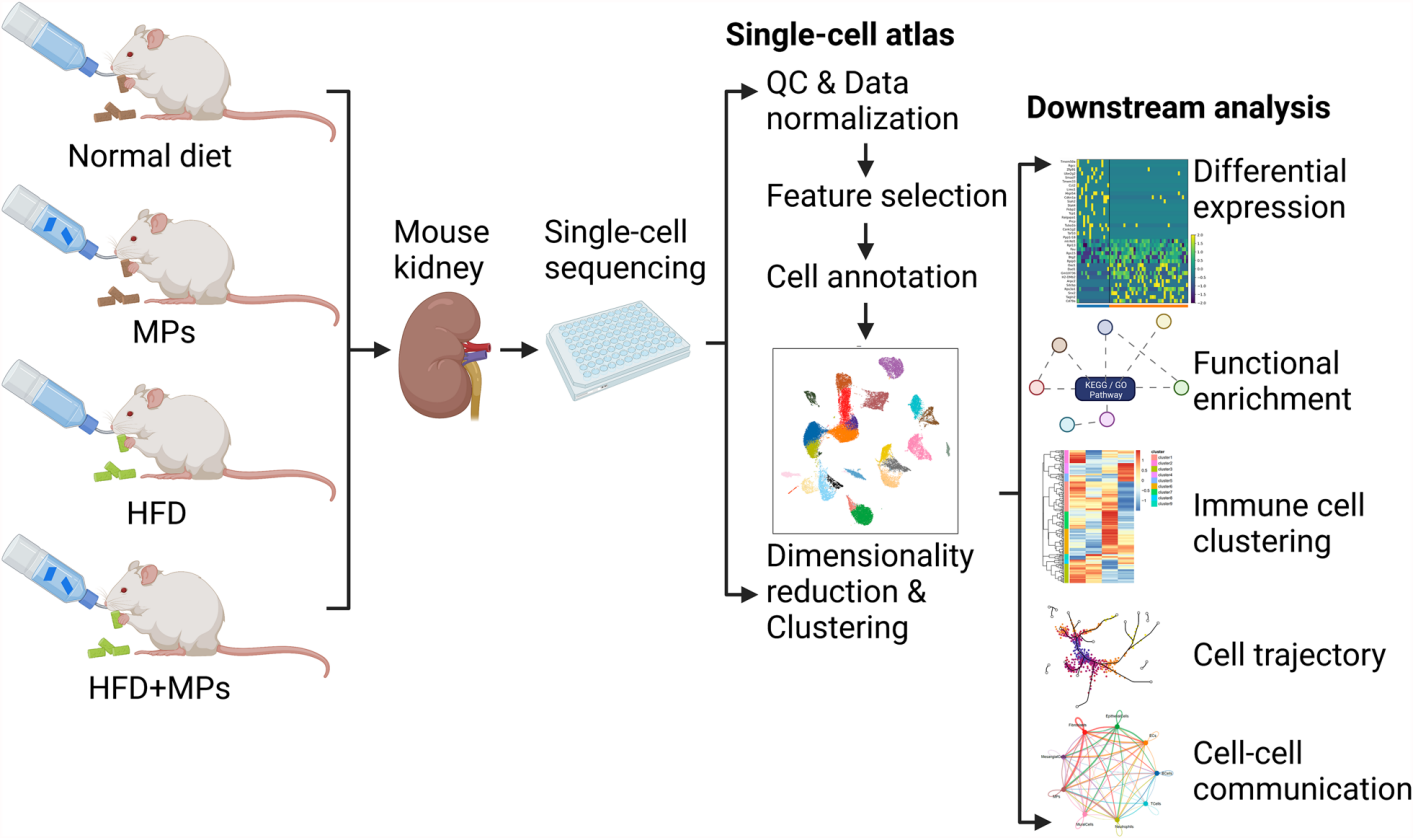

**Supplementary Information:**

The online version contains supplementary material available at 10.1186/s12951-023-02266-7.

## Introduction

The production and use of plastic substances have grown dramatically since the 1950s because of their physicochemical stability, degradation resistance, and low processing cost [1, 2]. The wide application of plastics in industrial production and daily life has brought great benefits and convenience to people. Still, around 174 million tons of plastics produced annually are also being released into the environment by humans [3]. Plastic pollution has aroused extensive concern in the world, especially microplastics. Microplastics are tiny plastic particles measuring less than 5 mm in diameter and have garnered considerable attention due to their potential environmental harm [4, 5]. They can be either intentionally manufactured at this size (primary microplastics), or they can result from the breakdown of larger plastic items (secondary microplastics) [6]. The growing industrial development and accumulation of plastic waste have led to the widespread distribution of microplastics across different environmental compartments globally, and extensive research has demonstrated pervasive microplastics in various environments worldwide [7]. It has brought a growing concern due to their potential harm to ecosystems [6, 8].

Due to their tiny and biorefractory nature, microplastics are easily absorbed by the food chain and accumulate in the tissues and organs of organisms over time through the bioaccumulation process [9]. Additionally, through biomagnification, the concentration of microplastics can increase at higher trophic levels, potentially affecting animals at the top of the food chain, including humans [10]. Microplastics can enter the human body via consumption and inhalation, so the air and food are the main sources of human microplastic uptake [11]. Microplastics can be present in fast food, takeout, beverages, and other high-fat diets (HFD) due to contamination during production, processing, packaging, and storage, accelerating the disruption of systemic metabolism [3, 12, 13]. Estimates of microplastic ingestion through HFD range from a few tens to hundreds of particles per day for humans [13, 14].

Polystyrene (PS) is widely used in food and makeup packaging, such as polyfoam, and it is easier to be mixed into food and absorbed by the human body. The accumulation of microplastic has been reported in various aquatic organisms and mammals, leading to a series of adverse effects. Some studies have revealed that exposure to accumulated toxins from ingested polystyrene microplastic (PS-MPs) can result in hepatic lipid disorder, renal dysfunction, intestinal dysbacteriosis, and endocrine disruption [12, 15, 16]. Notably, recent studies suggested that PS-MPs particle exposure through oral administration significantly increased oxidative stress, inflammation responses, and structural injury in the kidneys [17–19]. These findings prove that exposure to microplastics can lead to kidney injury in mice and contribute to our understanding of the potential risks associated with microplastic exposure on kidney health [20]. Following the discovery of microplastic particles in human blood, lungs, and placenta, Antonio Ragusa et al*.* found the presence of microplastic particles in human breastmilk in 2022, further confirming that ubiquitous microplastics presence makes human exposure inevitable [6, 21–23]. Therefore, understanding the potential health risks associated with microplastic exposure is essential for safeguarding human health, promoting risk assessment, and raising awareness among the general public. Previous studies showed that changes in diet altered metabolites, gene expression of nutrient transporters, and inflammatory cells and promoted carcinogenesis in mice [24–27]. Evaluating the kidney in studying endocrine and metabolic pathophysiology through the synergistic effects of PS-MPs and HFD is essential.

The effects of microplastics on the tissue immune microenvironment are an emerging area of research [28]. Prolonged exposure to PS-MPs has been shown to interact with immune cells, promoting the release of pro-inflammatory molecules, the generation of immunomodulatory reactive oxygen species (ROS), and dysregulation in immune cell populations [29–31]. Besides, PS-MPs can physically obstruct immune cells or interfere with their phagocytic activity, affecting their ability to clear pathogens or debris [32]. This impairment in immune cell function may compromise the tissue immune microenvironment and increase susceptibility to infections or other immune-related disorders [33]. Further research is needed to fully understand the mechanisms underlying the effects of microplastics on the tissue immune microenvironment and their potential implications for human health. Here, utilizing a single-cell transcriptomic sequencing approach, we compared the differences among kidney samples exposed to PS-MPs or HFD components in mice, thus further elucidating the heterogeneity of renal cells and unveiling the complex interactions between microplastics and the immune system dysregulation within kidney tissues.

## Material and methods

### In vivo models

A total of 16 six-week-old C57BL6 male mice were obtained from Shanghai Experimental Animal Center (Shanghai, China; ID: SCXK2007-0005). The mice were raised in a standard environment with a 12-h light/dark cycle and 50 ± 5% humidity at 20 ± 2 °C. After a week of acclimation, the mice were randomized into the following four groups of 4 mice each: normal diet (ND) group (normal diet for 18 weeks), PS-MPs group (fed with water containing 10 mg/L of 1 μm PS-MPs for 18 weeks), HFD group (fed with HFD [comprising 60% kilojoules of fat] for 18 weeks), and HFD plus PS-MPs group (fed with HFD and water containing 10 mg/L of 1 μm PS-MPs for 18 weeks). The amount of food consumed and changes in mice’s body weight were recorded weekly throughout the research. After 18 weeks, the mice were euthanized via deep anesthesia, and the bilateral kidneys were resected and weighed. All samples were then stored until further use.

PS-MPs were purchased from the Tianjin Baseline ChromTech Research Center (Tianjin, China). The shape of the particles was spherical, and their diameter was 1 µm. Because the physicochemical properties of MPs (e.g., shape, size, concentrations, surface charge, and hydrophobicity) affect the transformation, interaction, fate and bioavailability to organisms, the PS-MPs we chose were in accordance with previous reports [30, 34, 35].

### Photoacoustic imaging (PAI)

To acquire in vivo and ex vivo fluorescent images of mouse kidney exposed to PS-MPs, PAI was operated in the first near-infrared (NIR-I) region of the visible (400–700 nm) by using a UV/Vis/NIR spectrometer (Lambda 9, Perkin Elmer, Waltham, MA, USA), according to the previous literature with slight modifications [36, 37]. Briefly, the size and zeta potential of PS-MPs were measured by using dynamic light scattering (DLS) (Malver Nano-ZS 90; Malvern Instruments, Malvern, UK). Mice were exposed to PS-MPs (λ Ex: 620 nm, λ Em: 680 nm, Aladdin, #M120393) or saline for 24 h, and then were anesthetized and maintained on 1.5% isoflurane delivered by nose cone. Photoacoustic signals of PS-MPs localization in mouse kidney were verified by using NIR-I (500–700 nm) fluorescence imaging.

### Single-cell preparation

The kidney samples of mice in different feeding groups were collected for the scRNA-seq assay. Briefly, the kidney tissues were preserved in sCelLiveTM tissue preservation solution (Singleron Biotechnologies, Nanjing, China). Then, the tissues were separated into single-cell suspensions using Singleron Python Python™ Automated Tissue Separator (Singleron Biotechnologies) and sCelLive™ Tissue Isolation Mix (Singleron Biotechnologies) following the manufacturer’s instructions. Finally, cell viability was assessed under a microscope after staining with Trypan Blue (Sigma).

### Construction of scRNA-seq library

The single-cell suspensions described above were diluted with PBS (HyClone) to a concentration of 1 × 10^5^ cells/mL and placed on a microfluidic device. Then, scRNA-seq libraries were constructed with the GEXSCOPE® Single-Cell RNA Library Kit (Singleron Biotechnologies) and Singleron Matrix® Automated single-cell processing system (Singleron Biotechnologies) according to the Singleron GEXSCOPE® protocol. After the libraries were constructed, they were diluted to a concentration of 4 ng/μL and then combined and sequenced using the Illumina HiSeq X system (Illumina, San Diego, USA) to generate paired-end reads with a length of 150 base pairs.

### Processing and analysis of scRNA-seq data

The raw reads generated from sequencing were processed with a customized pipeline to obtain gene expression matrices. Quality control was performed using fastQC (version 0.11.4) (https://www.bioinformatics.babraham.ac.uk/projects/fastqc/) and fastp [38] to remove low-quality reads, and cutadapt was utilized for trimming poly-A tail and adapter sequences [39]. Cell barcodes and UMIs were then obtained from the reads. Subsequently, STAR software (version 2.5.3a) was applied to align the reads to the GRCm38 (mm10) reference genome. The feature Counts software (version 1.6.2) was used to obtain UMI counts and gene counts per cell, which was then utilized to generate expression profiles for further analyses. Before the assessments, cells with UMI counts < 30,000 and gene counts ranging from 200 to 5,000 were filtered, and cells with mitochondrial content > 20% were removed. Following this, dimensionality reduction and clustering were performed with the Seurat package in R software (version 3.1.2) [40]. A resolution of 1.2 was set for the sub-clustering of specific cell types within a cluster. Finally, the t-SNE or the UMAP algorithm was conducted to visualize cell subpopulations in a two-dimensional space.

### Functional enrichment analysis

Gene Ontology (GO) annotation and Kyoto Encyclopedia of Genes and Genomes (KEGG) pathway enrichment analyses were conducted with the “clusterProfiler” package in R software to identify distinct biological functions and pathways [41]. Gene Set Variation Analysis (GSVA) was performed to determine further the underlying biological functions of significant genes [42].

### Hematoxylin and eosin (HE) and Sirius Red staining

HE and Sirius Red staining were implemented to assess nephron morphology and collagen fibers in tissue sections. We randomly selected ten tissue slices procured from each group for the subsequent experiments. For HE analysis, tissues as indicated were harvested, fixed with 4% paraformaldehyde in PBS, embedded into paraffin blocks, sectioned (5 µm) and then stained with HE (Sigma) following standard protocol as previously described [43]. Sirius Red staining involves deparaffinization and rehydration of tissue slices, followed by incubation in Sirius Red solution for approximately an hour. Slices are then rinsed, differentiated in acidified water, dehydrated, and mounted with coverslips. Collagen fibers are visualized under a microscope, with polarized light revealing their characteristic birefringence, aiding in tissue analysis. Measurements and evaluation of histological activity scores including glomerular damage, tubular damage, and tubulointerstitial fibrosis were evaluated according to the previous literature with slight modifications [44, 45].

### Immunohistochemistry (IHC) and multispectral fluorescent IHC (mIHC) assays

IHC analysis was performed to assess the expression level of PF4 (ab303494; Abcam) in clear cell renal cell carcinoma (ccRCC) samples from Fudan University Shanghai Cancer Center (FUSCC) following manufacturers’ protocols as previously described [46]. The mIHC staining assay was conducted to investigate the abundance and distribution of PF4 (ab303494; Abcam), CD68 (ab125212; Abcam), and CD163 (25121; CST) in ccRCC adjacent normal kidney tissues following manufacturers’ protocols. Tissue slides that were bound with primary and secondary antibodies but not fluorophores were included as negative controls to assess autofluorescence. Multiplex stained slides were scanned using a Vectra Polaris Quantitative Pathology Imaging System (Akoya Biosciences) at 20 nm wavelength intervals from 440 to 780 nm with a fixed exposure time and an absolute magnification of 200×. All scans for each slide were then superimposed to obtain a single image. Multilayer images were imported to inForm v.2.4.8 (Akoya Biosciences) for quantitative image analysis.

### Statistical analysis

Data are represented as the mean ± standard deviation. To compare the two groups, the Student’s t-test (two-tailed) was used for normally distributed data, and the nonparametric Mann–Whitney test was used for non-normally distributed data. To compare three or more groups of normally distributed data, one-way analysis of variance (ANOVA) with post hoc Tukey’s multiple comparison test and two-way ANOVA with Šídák’s multiple comparisons test were used. The Kruskal–Wallis one-way ANOVA with Dunn’s multiple comparison test was used to compare non-normally distributed data. Different lowercase letters represent statistical significance by one-way ANOVA with Tukey’s post hoc test. The statistical significance was considered when *p*-values less than 0.05.

## Results

### Single-cell transcriptome landscape of mouse kidney altered by PS-MPs plus HFD feeding

**Fig. 1:**
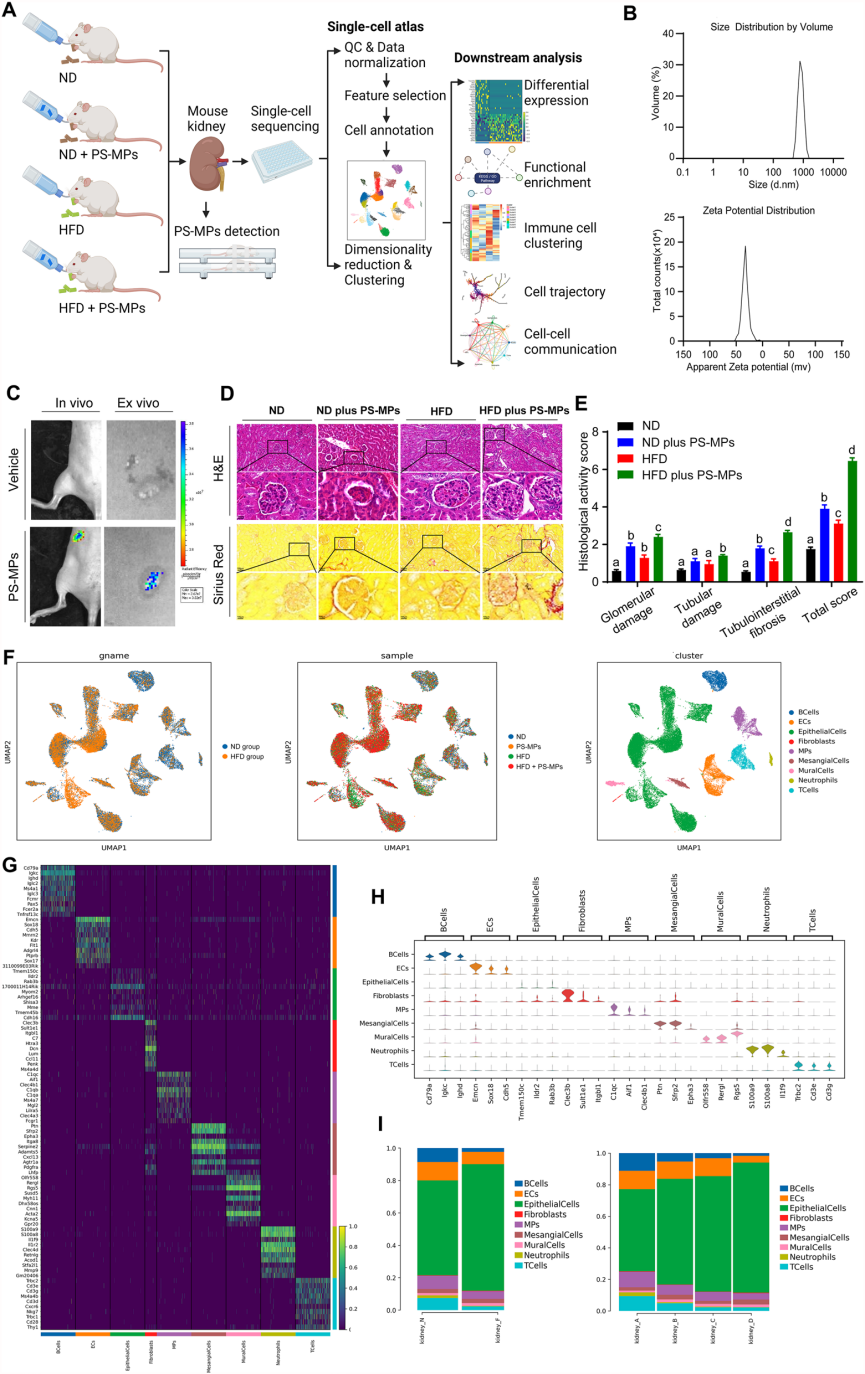
Comprehensive landscape of the impact of PS-MPs and HFD on renal tissues. **A** Scheme of mice exposure, kidney collection, cell suspension treatment, single-cell isolation, and scRNA-seq analysis. **B** The size and zeta potential of PS-MPs detected in mice. **C** Detecting the PS-MPs in mice utilizing near-infrared (NIR) window optical imaging technique. **D** HE and Sirius Red staining of renal tissues. **E** Histological activity scores of glomerular damage, tubular damage, and tubulointerstitial fibrosis in four groups. Different lowercase letters represent statistical significance by one-way ANOVA with Tukey’s post hoc test. **F** Uniform manifold approximation and projection (UMAP) of renal cells colored by distinct cell types. **G** Heatmap of the top 10 DEGs among all cell types according to log fold changes. **H** Violin plot of the top 3 DEGs among all cell types according to log fold changes. **I** The proportion of cell subpopulations in the ND and HFD groups (left) and four treatment groups (right)

In this study, we performed comprehensive research methods involving mouse exposure, kidney collection, cell suspension preparation, single-cell isolation, scRNA-seq analysis, and further downstream analysis to gain deep insight into the complicated effects of PS-MPs and HFD on the kidney. The study scheme was depicted in Fig. 1A. Figure 1B, C demonstrated that PS-MPs ingested by mice could be detected and measured. We conducted HE and Sirius Red staining for the four groups to explore the effects of PS-MPs and HFD on the mouse kidney. The results revealed that the treatment of PS-MPs plus HFD exacerbated glomerular damage, tubular damage, and tubulointerstitial fibrosis, which stimulated our curiosity to elucidate further the mechanism (Fig. 1D, E and Additional file 1; Table S1). After sample preparation and quality control, a total of 38,676 cells (13,369 cells from the ND group, 10,178 cells from the PS-MPs group, 7136 cells from the HFD group, and 7993 cells from the HFD plus PS-MPS group) were isolated and used for further scRNA-seq analysis (Fig. 1F). We distinguished and characterized nine cell types by unsupervised clustering analysis, including epithelial cells (25,605 cells, 66.20%), endothelial cells (3831 cells, 9.91%), mononuclear phagocytes (2661 cells, 6.88%), B cells (2381 cells, 6.16%), T cells (2101 cells, 5.43%), mesangial cells (976 cells, 2.52%), neutrophils (440 cells, 1.14%), and fibroblasts (102 cells, 0.26%) (Fig. 1F). The top 10 and top 3 differentially expressed genes (DEGs) in all cell types were displayed in Fig. 1G, H, respectively, which showed distinct transcriptome features of the nine cell types. Furthermore, we analyzed the proportion changes of each cell subpopulation in the mouse kidney after different treatments. The results demonstrated that the proportions of B, T, and endothelial cells were reduced. In contrast, proportions of epithelial cells and mononuclear phagocytes increased in both PS-MPs and HFD groups, and the above effects were amplified in the PS-MPs plus HFD group (Fig. 1I and Additional file 1; Table S2).

### Changes of subsets and biological features in renal epithelial cells after PS-MPs plus HFD treatment

**Fig. 2:**
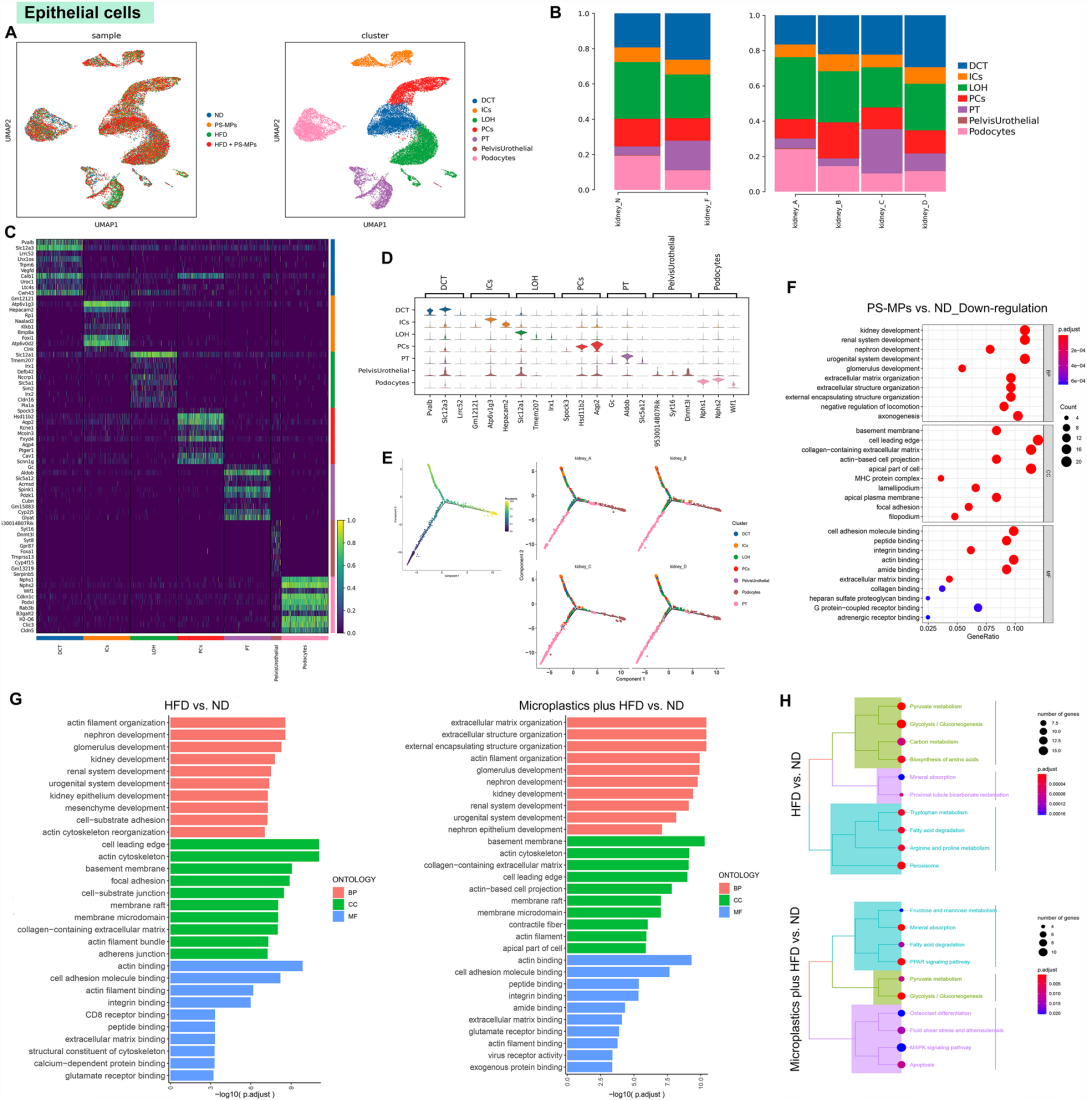
The development of renal epithelial cells was suppressed by PS-MPs plus HFD treatment. **A** UMAP of epithelial cells colored by four treatment groups (left) and epithelial cell subsets (right). **B** The proportion of epithelial cell subsets in the ND and HFD groups (left) and four treatment groups (right). **C** Heatmap of the top 10 DEGs among epithelial cell subsets according to log fold changes. **D** Violin plot of the top 3 DEGs among epithelial cell subsets according to log fold changes. **E** Pseudotime trajectory analysis colored by epithelial cell subsets at varying pseudotime stages (left) and by individual epithelial cell subsets (right). **F** GO enrichment analysis of down-regulation pathways of epithelial cells in PS-MPs group vs. ND group. **G** GO enrichment analysis of up-regulation pathways of epithelial cells in HFD group vs. ND group (left) and PS-MPs plus HFD group vs. ND group (right). **H** KEGG enrichment analysis of upregulation pathways of epithelial cells in HFD group vs. ND group (up) and PS-MPs plus HFD group vs. ND group (down)

Considering the predominance of epithelial cells within the mouse kidney and their reduction following treatment with PS-MPs plus HFD, we first focused on investigating the impact of this treatment on epithelial cells. Unsupervised clustering analysis of 25,605 epithelial cells identified seven distinct subsets, including distal convoluted tubule (DCT) cells, collecting duct intercalated cells (ICs), kidney loop of Henle epithelial cells (LOH), Collecting duct principal cells (PCs), proximal tubule (PT) cells, kidney pelvis urothelial cells (PelvisUrothelial), and podocytes (Fig. 2A). Upon PS-MPs treatment, mouse kidneys, whether on ND or HFD group, exhibited increased proportions of DCT cells, ICs, and PCs, while the proportion of PT cells decreased (Fig. 2B and Additional file 1; Table S2). Subsequent analysis of DEGs unveiled significant variations in transcription levels among the epithelial cell subpopulations (Fig. 2C, D). Moreover, we employed pseudotime analysis to elucidate the differentiation trajectory from PT cells to podocytes (Fig. 2E). Intriguingly, the differentiation trend was regressed after PS-MPs plus HFD treatment, suggesting a potential inhibitory effect on the development of renal epithelial cells (Fig. 2E). We performed functional enrichment analysis within the specific groups to further explore the underlying impact of PS-MPs plus HFD on epithelial cells. GO analysis revealed that kidney development-related pathways were significantly down-regulated after PS-MPs treatment, including nephron development, glomerulus development, renal system development, and kidney epithelium development (Fig. 2F). Remarkably, these pathways were also enriched in the HFD group and PS-MPs plus HFD group, further suggesting an inhibitory role of PS-MPs plus HFD in the developing of renal epithelial cells (Fig. 2G). Additionally, KEGG analysis unveiled up-regulation of the MAPK signaling pathway, glycolysis pathway, apoptosis pathway, and PPAR signaling pathway after treatment with PS-MPs plus HFD (Fig. 2H). These findings may have implications for understanding the molecular mechanisms underlying epithelial cell responses to PS-MPs plus HFD treatment, thereby warranting further investigations to unravel the detailed regulatory networks involved.

### Cellular dynamics and functional changes in PT and DCT cells under PS-MPs plus HFD treatment

**Fig. 3:**
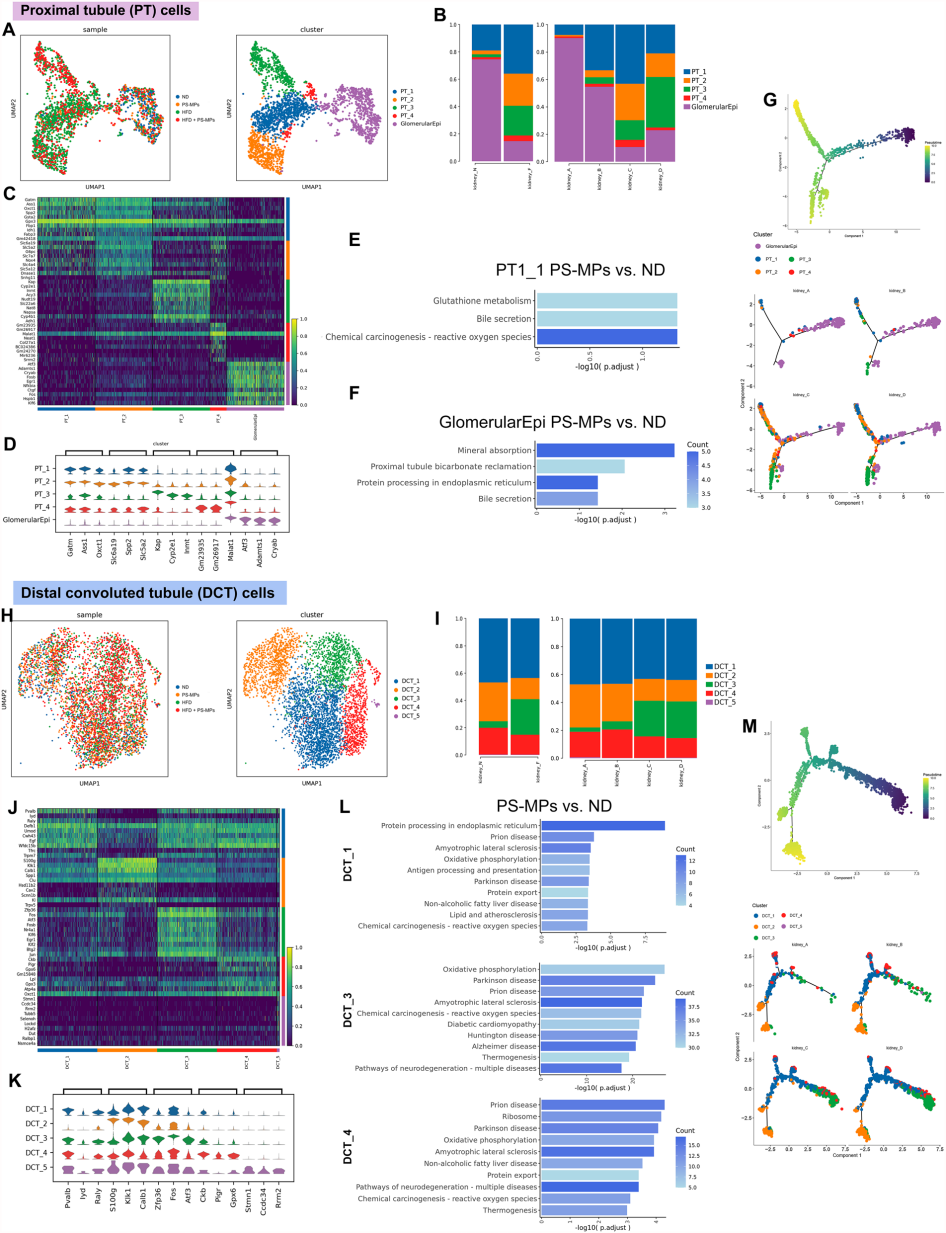
Changes of cell subsets proportions and biological features in PT and DCT cells upon PS-MPs plus HFD treatment. **A** UMAP of PT cells colored by four treatment groups (left) and PT cell subsets (right). **B** The proportion of PT cell subsets in the ND and HFD groups (left) and four treatment groups (right). **C** Heatmap of the top 10 DEGs among PT cell subsets according to log fold changes. **D** Violin plot of the top 3 DEGs among PT cell subsets according to log fold changes. **E** KEGG enrichment analysis of upregulation pathways of PT1_1 cells in PS-MPs group vs. ND group. **F** KEGG enrichment analysis of upregulation pathways of GlomerularEpi cells in PS-MPs group vs. ND group. **G** Pseudotime trajectory analysis colored by PT cell subsets at varying pseudotime stages (up) and by individual PT cell subsets (down). **H** UMAP of DCT cells colored by four treatment groups (left) and PT cell subsets (right). **I** The proportion of DCT cell subsets in the ND and HFD groups (left) and four treatment groups (right). **J** Heatmap of the top 10 DEGs among DCT cell subsets according to log fold changes. **K** Violin plot of the top 3 DEGs among DCT cell subsets according to log fold changes. **L** KEGG enrichment analysis of upregulation pathways of DCT_1, DCT_3, and DCT_4 in PS-MPs group vs. ND group. **M** Pseudotime trajectory analysis colored by DCT cell subsets at varying pseudotime stages (up) and individual DCT cell subsets (down)

Next, we investigated the effects of PS-MPs plus HFD on PT and DCT cells within the epithelial cells. Sub-clustering of 2474 PT cells revealed five distinct clusters, including PT_1, PT_2, PT_3, PT_4, and glomerular epithelial cells (GlomerularEpi) (Fig. 3A). Following treatment with PS-MPs, we observed significant changes in the composition of PT cell subsets. Specifically, there was a notable increase in the abundance of PT_1 and PT_3, which initially exhibited low content. Conversely, GlomerularEpi, the major cell subset, significantly reduced after PS-MPs treatment (Fig. 3B and Additional file 1; Table S2). The most significant DEGs among PT cell subsets were visually depicted in Fig. 3C, D. Considering the most pronounced changes in the abundance of PT_1 and glomerular epithelial cells following PS-MPs treatment, we performed functional enrichment analysis to explore their distinctive functional characteristics. KEGG pathway analysis revealed activation of chemical carcinogenesis-reactive oxygen species and glutathione metabolism pathways in PT_1. In contrast, after PS-MPs treatment, mineral absorption, and protein processing pathways were up-regulated in glomerular epithelial cells (Fig. 3E, F). Pseudotime analysis revealed that PT cell differentiation predominantly followed a trajectory originating from GlomerularEpi and sequentially transitioning into the other four clusters. Upon intervention with PS-MPs plus HFD, there was a marked amplification in the proportion of GlomerularEpi cells transitioning to the other four clusters (Fig. 3G). Moreover, we performed sub-clustering analysis on 4594 DCT cells, which revealed the existence of five distinct clusters, denoted as DCT_1, DCT_2, DCT_3, DCT_4, and DCT_5 (with scarce content) (Fig. 3H). Upon treatment with PS-MPs, there was a reduction in the proportions of DCT_1 and DCT_2, while the compositions of DCT_3 and DCT_4 increased (Fig. 3I and Additional file 1; Table S2). The identified DEGs among DCT cell subsets are illustrated in Fig. 3J, K. Remarkably, after PS-MPs treatment, DCT_1, DCT_3, and DCT_4 subgroups exhibited significant activation of pathways associated with chemical carcinogenesis-reactive oxygen species, oxidative phosphorylation, and neurodegenerative diseases. This observation suggests that PS-MPs may play an essential role in the pathogenesis of these disease-related pathways (Fig. 3L). The results of pseudotime analysis revealed that DCT_3 represented the early differentiation stage of DCT cells. Treatment with PS-MPs plus HFD appeared to impede the normal differentiation trajectory of DCT cells, indicating a potential inhibitory effect on their development (Fig. 3M).

### Impact of PS-MPs plus HFD on the biological characteristics and differentiation trajectories of ECs

**Fig. 4:**
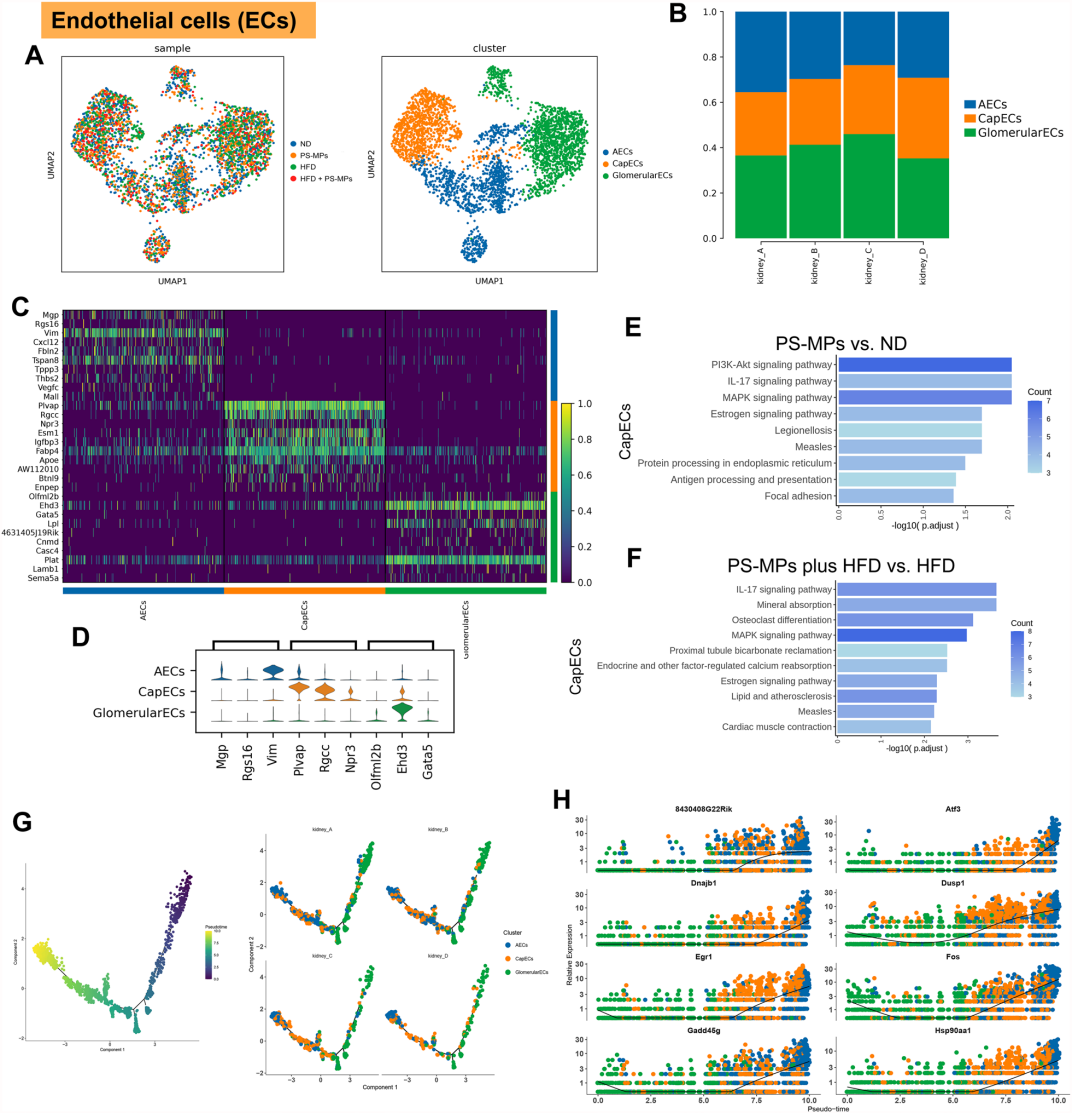
Changes in the biological features and differentiation trajectories of ECs upon PS-MPs plus HFD treatment. **A** UMAP of endothelial cells colored by four treatment groups (left) and PT cell subsets (right). **B** The proportion of endothelial cell subsets in the four treatment groups. **C** Heatmap of the top 10 DEGs among endothelial cell subsets according to log fold changes. **D** Violin plot of the top 3 DEGs among endothelial cell subsets according to log fold changes. **E** KEGG enrichment analysis of upregulation pathways of CapECs in PS-MPs group vs. ND group. **F** KEGG enrichment analysis of upregulation pathways of CapECs in PS-MPs plus HFD group vs. HFD group. **G** Pseudotime trajectory analysis colored by endothelial cell subsets at varying pseudotime stages (left) and by individual endothelial cell subsets (right). **H** The top 8 differential expression genes with pseudotime progression

To further elucidate the effects of PS-MPs plus HFD on stromal compositions within the renal microenvironment, we conducted a comprehensive investigation focusing on ECs. Sub-clustering of 3683 ECs using unsupervised clustering analysis revealed three distinct clusters, namely arterial endothelial cells (AECs), capillary endothelial cells (CapECs), and glomerular endothelial cells (GlomerularECs) (Fig. 4A). Upon treatment with PS-MPs, the proportion of GlomerularECs increased, while the proportion of AECs decreased. Remarkably, PS-MPs plus HFD displayed a synergistic effect, increasing the abundance of CapECs (Fig. 4B and Additional file 1; Table S2). The top DEGs among EC subsets were visually displayed in Fig. 4C, D. Furthermore, we performed an enrichment analysis to explore the functional characteristics of CapECs. Notably, in the PS-MPs treated ND group, the PI3K-Akt signaling pathway, IL-17 signaling pathway, and MAPK signaling pathway were up-regulated (Fig. 4E). Meanwhile, after PS-MPs treatment, the HFD group showed an activation trend in the IL-17 signaling pathway, MAPK signaling pathway, and estrogen signaling pathway (Fig. 4F). Pseudotime analysis provided insights into the differentiation trajectory of ECs. The trajectory was observed to initiate from GlomerularECs, and PS-MPs plus HFD treatment induced the differentiation of other cell subsets (Fig. 4G). Additionally, the top 8 genes exhibiting differential expression with pseudotime progression were depicted in Fig. 4H.

### Profiling the impact of PS-MPs and HFD on T cell and B cell subsets in the renal microenvironment

**Fig. 5:**
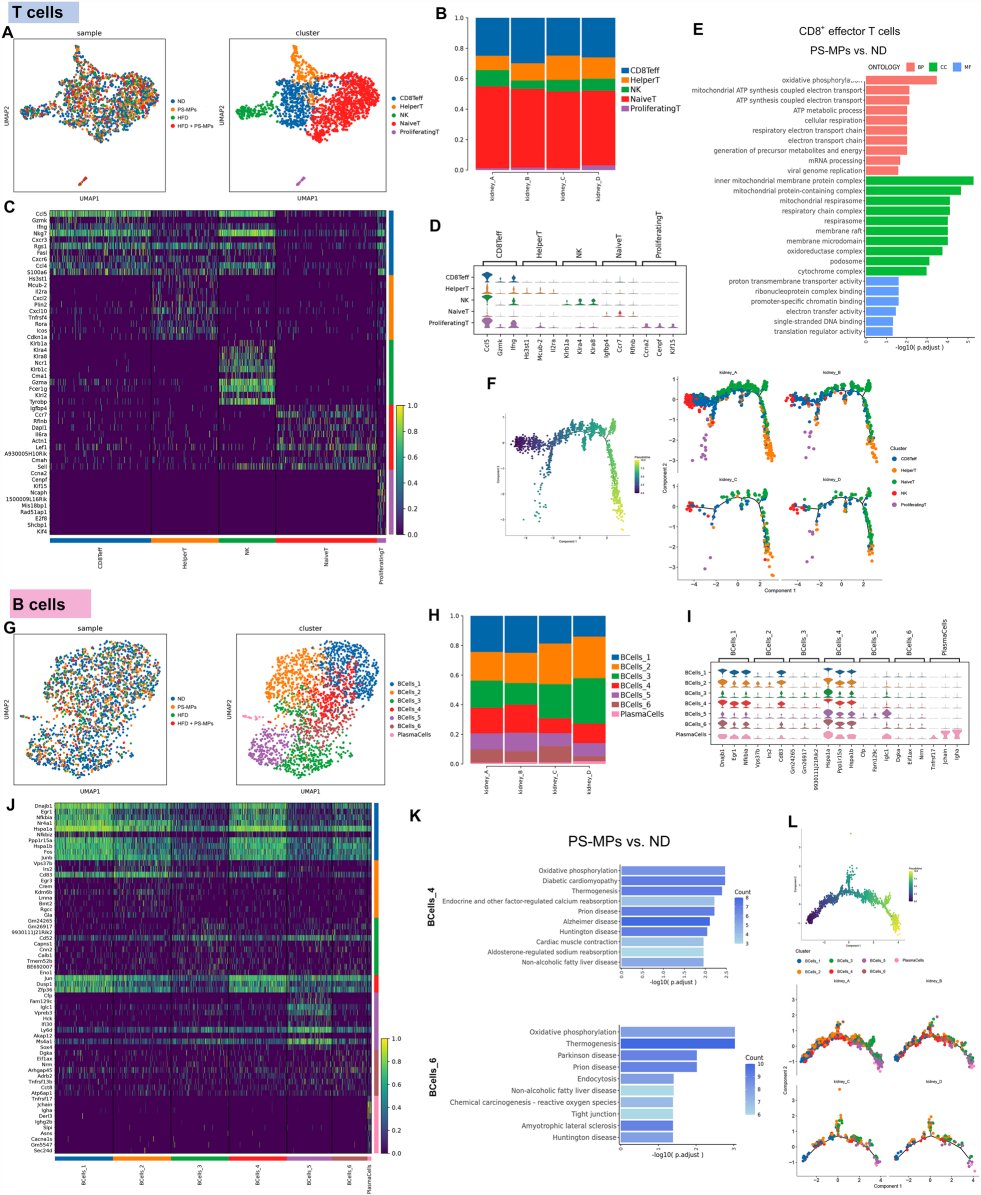
PS-MPs plus HFD treatment reshaped the proportions and activation states of T and B cells. **A** UMAP of T cells colored by four treatment groups (left) and T cell subsets (right). **B** The proportion of T cell subsets in the four treatment groups. **C** Heatmap of the top 10 DEGs among T cell subsets according to log fold changes. **D** Violin plot of the top 3 DEGs among T cell subsets according to log fold changes. **E** KEGG enrichment analysis of upregulation pathways of CD8^+^ effector T cells in PS-MPs group vs. ND group. **F** Pseudotime trajectory analysis colored by T cell subsets at varying pseudotime stages (left) and by individual T cell subsets (right). **G** UMAP of B cells colored by four treatment groups (left) and B cell subsets (right). **H** The proportion of B cell subsets in the four treatment groups. **I** Violin plot of the top 3 DEGs among B cell subsets according to log fold changes. **J** Heatmap of the top 10 DEGs among B cell subsets according to log fold changes. **K** KEGG enrichment analysis of upregulation pathways of BCells_4 and BCells_6 in PS-MPs group vs. ND group. **L** Pseudotime trajectory analysis colored by B cell subsets at varying pseudotime stages (up) and by individual B cell subsets (down)

In this section, we investigated the impact of PS-MPs and HFD on immune cell components, specifically T cells and B cells, within the renal microenvironment. Sub-clustering of 1867 T cells revealed five subsets, including CD8^+^ effector T cells (CD8Teff), T helper cells (HelperT), natural killer cells (NK), naive T cells (NaiveT), and proliferating T cells (ProliferatingT) (Fig. 5A). Notably, in both ND and HFD groups, there was a reduction in the proportion of NaiveT, while an increase in the abundance of CD8Teff and ProliferatingT following PS-MPs treatment (Fig. 5B and Additional file 1; Table S2). The top DEGs among T cell subsets were visually displayed in Fig. 5C, D. GO analysis of CD8Teff with PS-MPs treatment revealed up-regulation of pathways related to oxidative phosphorylation, inner mitochondrial membrane protein complex, and proton transmembrane transporter activity (Fig. 5E). The pseudotime analysis suggested that PS-MPs plus HFD treatment appeared to impede the maturation of NaïveT cells (Fig. 5F). Sub-clustering of 2147 B cells revealed seven subsets, including BCells_1, BCells_2, BCells_3, BCells_4, BCells_5, BCells_6, and plasma cells (PlasmaCells) (Fig. 5G). Notably, in both ND and HFD groups, the proportions of BCells_4 and plasma cells increased, while the proportion of BCells_6 decreased after PS-MPs treatment (Fig. 5H and Additional file 1; Table S2). Figure 5I, J displayed the significant DEGs among B cell subsets. Considering the rarity of plasma cells, we next focused on performing enrichment analysis for BCells_4 and BCells_6. For BCells_4, KEGG analysis revealed oxidative phosphorylation, thermogenesis, and neurodegenerative disease-related pathways were activated in the ND group after PS-MPs treatment. In BCells_6, treatment with PS-MPs activated oxidative phosphorylation, chemical carcinogenesis-reactive oxygen species, and thermogenesis-related pathways in the ND group (Fig. 5K). Pseudotime analysis provided insights into the progressive differentiation pattern within B cell subclusters, transitioning predominantly from BCells_1 and BCells_4 to the BCells_5 and PlasmaCells (Fig. 5L).

### Unraveling MPs’ diverse characteristics and functional dynamics within the renal microenvironment upon PS-MPs and HFD treatment

**Fig. 6:**
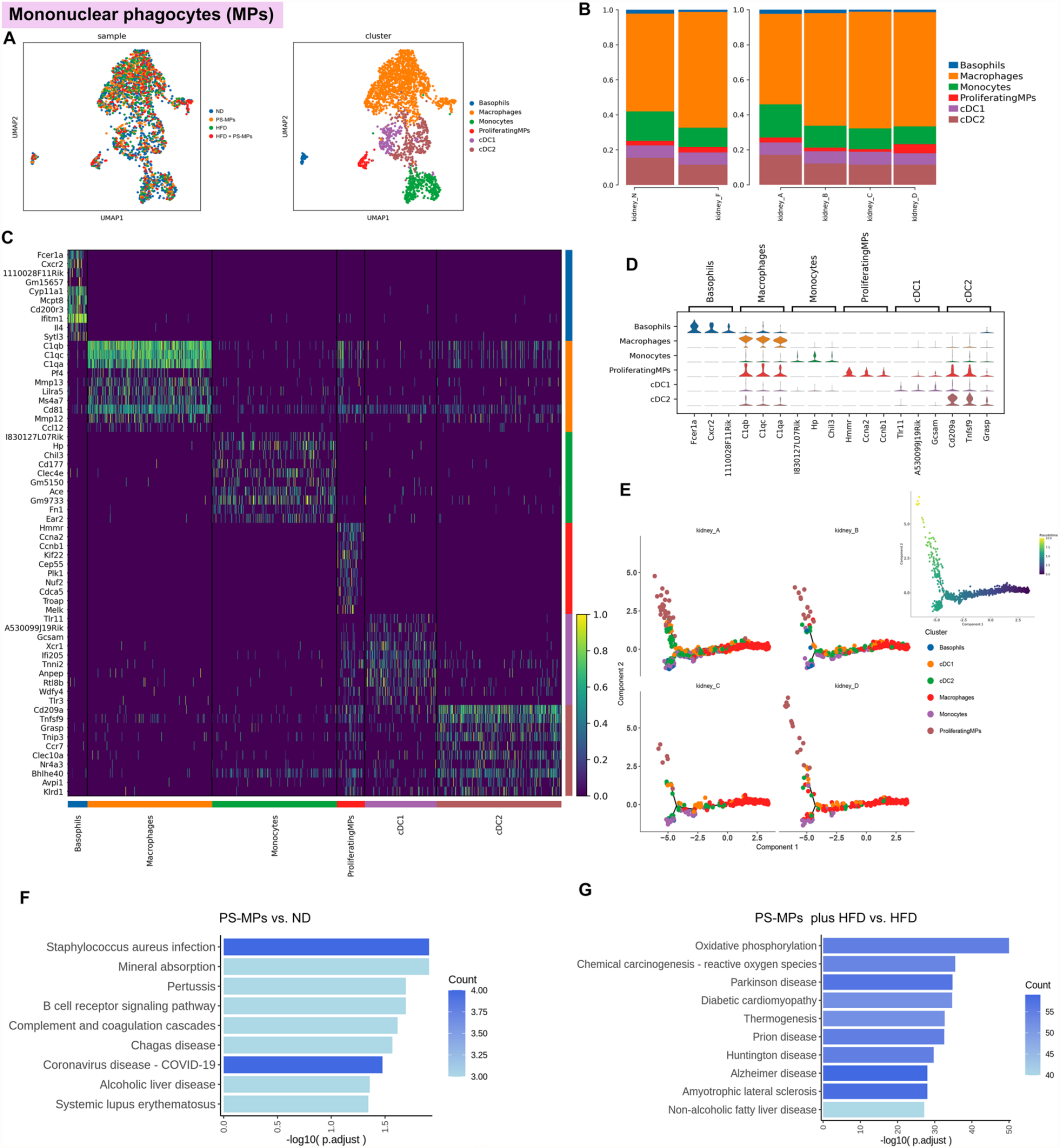
Effects of PS-MPs plus HFD treatment on cell subsets and biological pathways activation in MPs. **A** UMAP of mononuclear phagocytes colored by four treatment groups (left) and PT cell subsets (right). **B** The proportion of mononuclear phagocyte subsets in the ND and HFD groups (left) and four treatment groups (right). **C** Heatmap of the top 10 DEGs among mononuclear phagocyte subsets according to log fold changes. **D** Violin plot of the top 3 DEGs among mononuclear phagocyte subsets according to log fold changes. **E** Pseudotime trajectory analysis colored by mononuclear phagocyte subsets at varying pseudotime stages (right) and by individual mononuclear phagocyte subsets (left). **F** KEGG enrichment analysis of upregulation pathways of mononuclear phagocytes in PS-MPs group vs. ND group. **G** KEGG enrichment analysis of upregulation pathways of mononuclear phagocytes in PS-MPs plus HFD group vs. HFD group

Through unsupervised clustering analysis, we identified six distinct cell subsets among 2451 MPs, including basophils, macrophages, monocytes, proliferating mononuclear phagocytes (ProliferatingMPs), conventional type 1 dendritic cells (cDC1), and conventional type 2 dendritic cells (cDC2) (Fig. 6A). Following PS-MPs treatment, we observed an elevation in the abundance of macrophages, accompanied by a reduction in the proportions of basophils and ProliferatingMPs. Importantly, PS-MPs plus HFD exhibited a synergistic effect, leading to decreased monocytes and cDC2 (Fig. 6B and Additional file 1; Table S2). Figure 6C, D visually depicts the most significant DEGs among MPs subsets. The results of pseudotime analysis revealed that macrophages represented the early differentiation stage of MPs (Fig. 6E). Additionally, we performed an enrichment analysis of MPs in specific groups. After PS-MPs treatment, pathways related to staphylococcus aureus infection, coronavirus disease, B cell receptor signaling, and complement and coagulation cascades were activated in the ND group (Fig. 6F). The HFD group demonstrated an activation trend in oxidative phosphorylation, chemical carcinogenesis-reactive oxygen species, and neurodegenerative disease-related pathways following PS-MPs treatment (Fig. 6G).

### Heterogeneity and functional dynamics of macrophages in response to PS-MPs plus HFD treatment in the kidney microenvironment

**Fig. 7:**
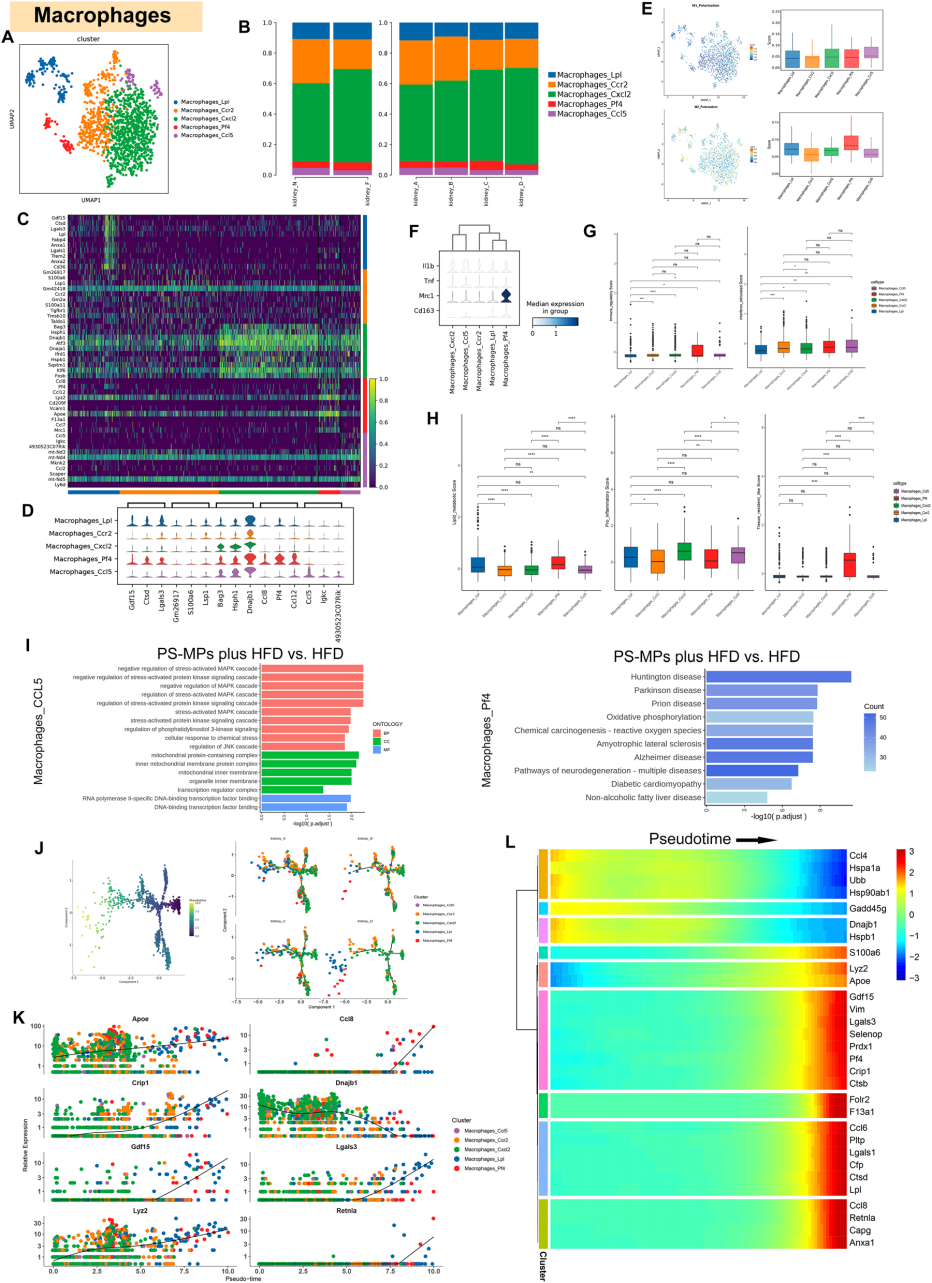
Macrophage subset alterations and polarization effects in kidney microenvironment under PS-MPs plus HFD treatment. **A** UMAP of macrophages colored by four treatment groups (left) and macrophage subsets (right). **B** The proportion of macrophage subsets in the ND and HFD groups (left) and four treatment groups (right). **C** Heatmap of the top 10 DEGs among macrophage subsets according to log fold changes. **D** Violin plot of the top 3 DEGs among macrophage subsets according to log fold changes. **E** The M1 and M2 polarization scores in distinct macrophage subsets. **F** Relative expression level of macrophage markers in distinct macrophage subsets. **G** The immune regulatory score and interferon-stimulated score in distinct macrophage subsets. **H** The lipid metabolic, pro-inflammatory, and tissue resident-like scores in distinct macrophage subsets. **I** GO enrichment analysis of upregulation pathways of Macrophages_CCL5 in PS-MPs plus HFD group vs. HFD group (left) and KEGG enrichment analysis of upregulation pathways of Macrophages_Pf4 in PS-MPs plus HFD group vs. HFD group (right). **J** Pseudotime trajectory analysis colored by macrophage subsets at varying pseudotime stages (right) and by individual macrophage subsets (left). **K** The top 8 differential expression genes with pseudotime progression among macrophage subsets. **L** Heatmap of differential expression genes with pseudotime progression

As the largest subsets within the MPs, we next aimed to investigate the alterations in macrophages within the kidney microenvironment upon treatment with PS-MPs plus HFD. Sub-clustering of 1437 macrophages revealed five distinct subsets, namely Macrophages_Lpl, Macrophages_Ccr2, Macrophages_Cxcl2, Macrophages_Pf4, and Macrophages_Ccl5 (Fig. 7A). Following PS-MPs treatment, NC and HFD groups displayed an increased abundance of Macrophages_Cxcl2, along with reduced proportions of Macrophages_Lpl and Macrophages_Pf4 (Fig. 7B and Additional file 1; Table S2). The most significant DEGs among macrophage subsets were visually illustrated in Fig. 7C, D. Next, we calculated the M1-macrophage and M2-macrophage polarization scores for each distinct subpopulation, revealing that Macrophages_Ccl5 exhibited characteristics similar to M1-macrophages, while Macrophages_Pf4 displayed similarities to M2-macrophages (Fig. 7E). Figure 7F depicts the expression levels of classical macrophage marker genes (M1-macrophage: IL1B, TNF; M2-macrophage: MRC1, CD163) across different subsets. Macrophages_Pf4 notably showed high expression of MRC1, indicative of M2-macrophage-like characteristics. To further explore macrophage heterogeneity, we evaluated the gene set scores of each subset. Remarkably, Macrophages_Pf4 exhibited the highest immune regulatory score, suggesting its role akin to M2-macrophages. In contrast, Macrophages_Ccl5 demonstrated elevated interferon-stimulated and pro-inflammatory scores, consistent with M1-macrophage functions. Additionally, Macrophages_Cxcl2 presented the highest pro-angiogenic score, while Macrophages_Lpl displayed the highest extracellular matrix (ECM) remodeling score (Fig. 7G, H). Furthermore, we conducted functional enrichment analyses to explore the impact of PS-MPs plus HFD treatment on Macrophages_Ccl5 and Macrophages_Pf4. GO analysis revealed activation of pathways related to MAPK cascade, PI3K cascade, and JNK cascade in Macrophages_Ccl5 after PS-MPs treatment (Fig. 7I). Similarly, KEGG analysis revealed up-regulation of neurodegenerative diseases, oxidative phosphorylation, and chemical carcinogenesis-reactive oxygen species pathways in Macrophages_Pf4 after PS-MPs treatment (Fig. 7I). Pseudotime analysis provided insights into the differentiation trajectory of macrophages, primarily initiating from Macrophages_Cxcl2, with PS-MPs plus HFD treatment inducing the differentiation of other macrophage subsets (Fig. 7J). Additionally, the top 8 genes exhibiting differential expression with pseudotime progression were visually depicted in Fig. 7K. Moreover, Fig. 7L demonstrated the division of macrophages into nine clusters based on genes expressed differentially over pseudotime.

### PF4^+^ macrophages enhanced renal fibrosis following PS-MPs plus HFD treatment

**Fig. 8:**
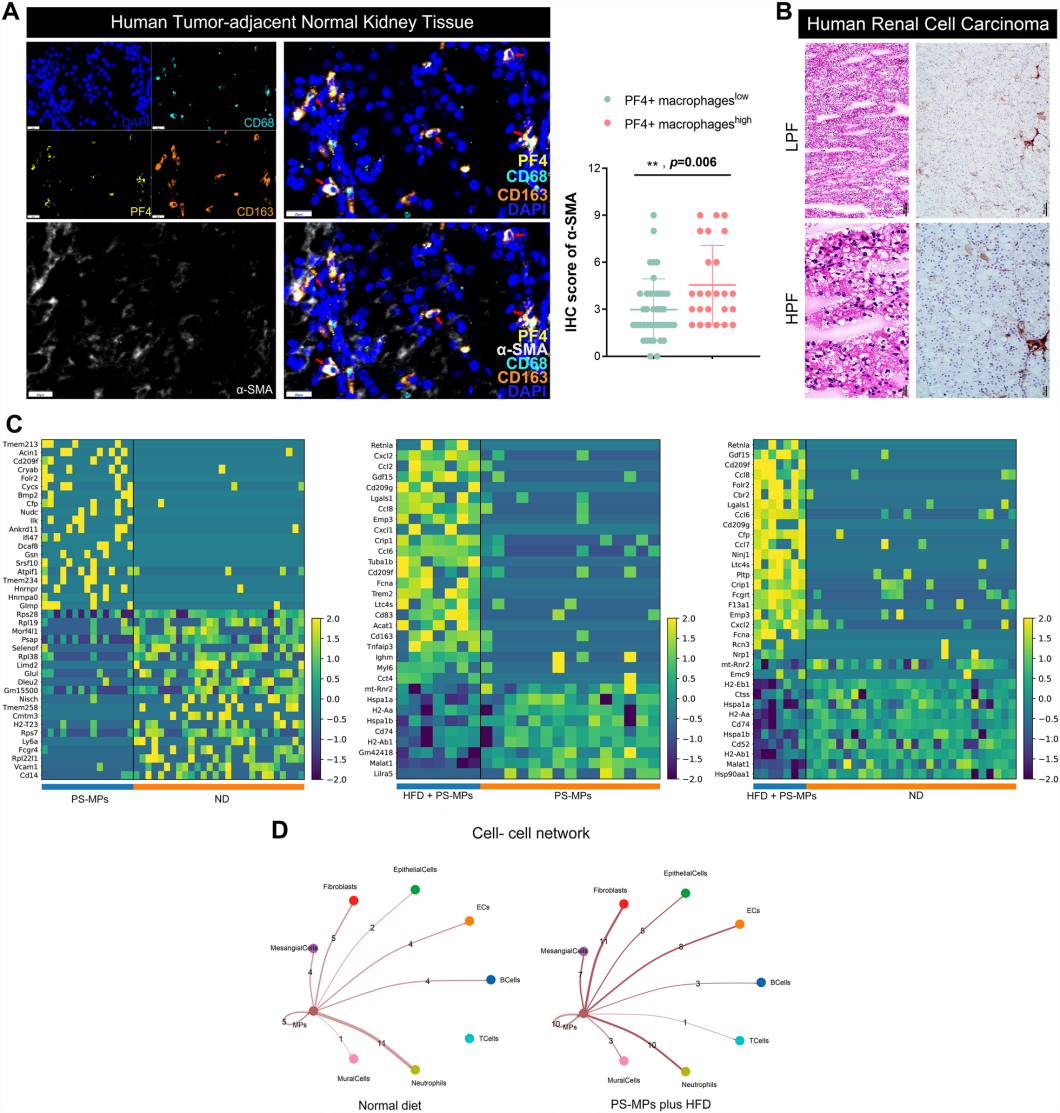
PF4^+^ macrophages enhanced renal fibrosis following PS-MPs plus HFD treatment. **A** mIHC staining of PF4, CD68, CD163, and α-SMA in ccRCC adjacent normal tissue (left) and IHC score of α-SMA between low and high expression of PF4^+^ macrophage groups. **B** IHC staining of PF4 in ccRCC tissue. **C** Heatmap of DEGs of Macrophages_Pf4 in four groups. **D** Interaction networks of intercellular communications between ND and PS-MPs plus HFD groups

Considering the striking resemblance of Macrophages_Pf4 to M2-macrophages, we investigated their functional characteristics in the context of ccRCC and adjacent normal tissues. Utilizing mIHC, we observed a substantial overlap between PF4^+^ macrophages and M2-macrophages (CD68^+^ CD163^+^) in adjacent normal tissues (Fig. 8A). Furthermore, the pronounced upregulation of α-SMA with the increase of PF4^+^ macrophage content provided compelling evidence of the potential involvement of PF4^+^ macrophages in renal fibrosis induction, consistent with previous reports [47] (Fig. 8A and Additional file 1; Table S3). IHC staining of human ccRCC tissues further confirmed the expression of PF4 in the tumor microenvironment (Fig. 8B). We analyzed the DEGs in Macrophages_Pf4 among the four groups to explore the underlying molecular mechanisms, revealing distinct gene expression patterns (Fig. 8C). Subsequently, we investigated the intercellular communications between MPs and fibroblasts after PS-MPs plus HFD treatment. Interestingly, the results indicated PS-MPs plus HFD treatment enhanced cell-to-cell communication between MPs and fibroblasts, suggesting that PF4^+^ macrophages may contribute to renal fibrosis by directly interacting with fibroblasts (Fig. 8D).

### Interaction landscape of cell subpopulations in kidney microenvironment after PS-MPs plus HFD treatment

**Fig. 9:**
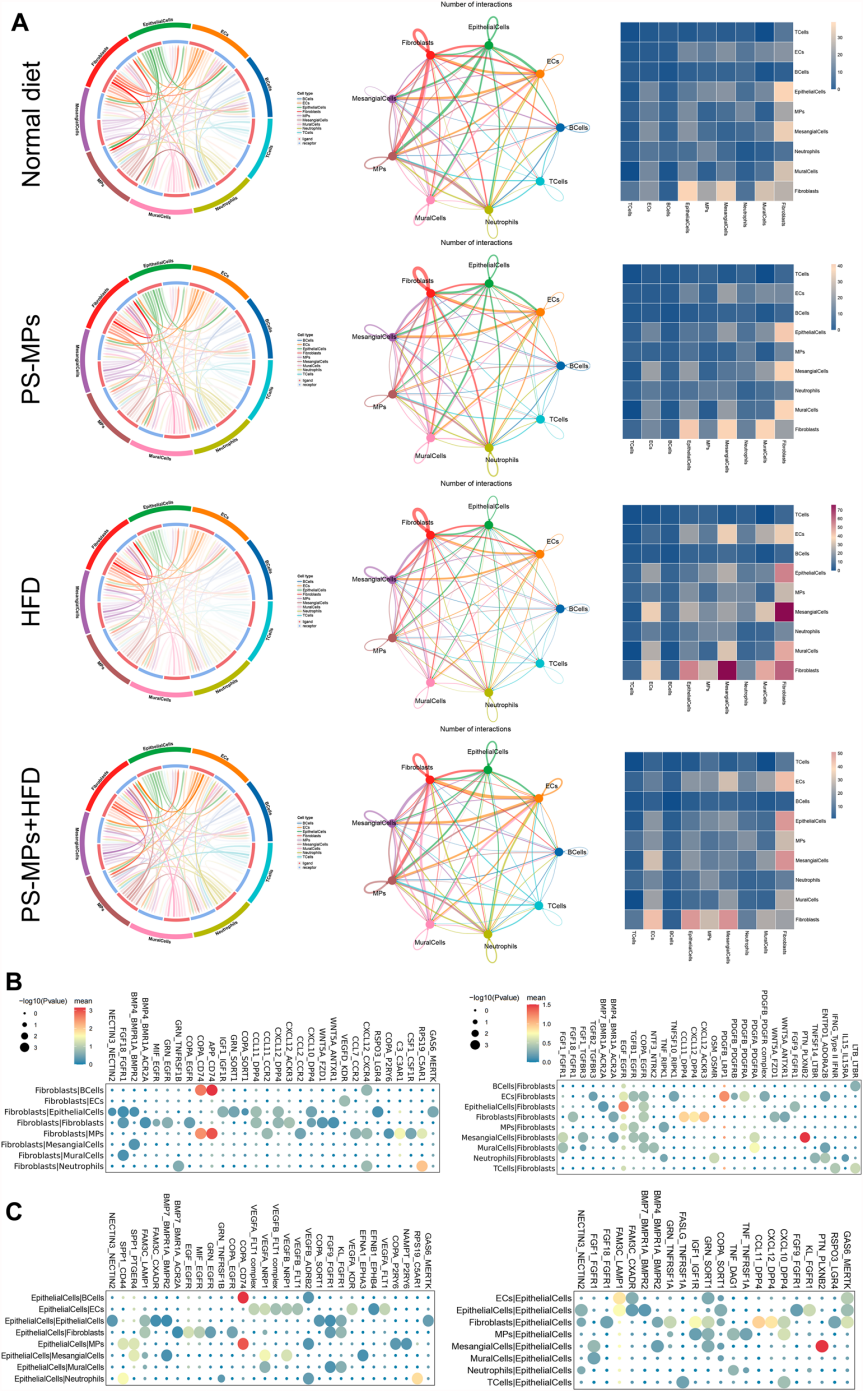
Interaction landscape of cell subpopulations in kidney microenvironment following PS-MPs plus HFD treatment. **A** Interaction networks of all cell subpopulations (left and middle), and heatmap of the amount of interaction gene pairs between two cell types (right), under the treatments of ND, PS-MPs, HFD, and PS-MPs plus HFD, respectively. **B** Bubble plot of interacting gene pairs between fibroblasts, acting as ligands (left) and receptors (right), with other cell types. **C** Bubble chart of interacting gene pairs between epithelial cells, acting as ligands (left) and receptors (right), with other cell types

To investigate the impact of PS-MPs plus HFD on the intercellular interactions within the kidney microenvironment, we employed interaction networks and heat maps to visualize the intensity of these interactions. Our findings demonstrated that fibroblasts were pivotal in mediating intercellular communications following the treatment with MPs plus HFD (Fig. 9A). Notably, when fibroblasts acted as ligand cells, they exhibited significant interaction gene pairs with other cell types, including APP-CD74, COPA-CD74, RPS19-C5AR1, and C3-C3AR1. Conversely, in the role of recipient cells, fibroblasts displayed notable interaction gene pairs, such as PTN-PLXNB2, EGF-EFGR, COPA-EGFR, and PDGFB-LRP1 (Fig. 9B). Epithelial cells, the most abundant subpopulation in the kidney microenvironment, demonstrated robust intercellular interactions with other cell types. As ligand cells, epithelial cells exhibited strong interaction gene pairs, including COPA-CD74, RPS19-C5AR1, and SPP1-CD44. In the role of recipient cells, epithelial cells displayed significant interaction gene pairs, such as PTN-PLXNB2, FAM3C-LAMP1, CXCL12-DPP4, and CCL11-DPP4 (Fig. 9C).

### Characterization of distinct mural cell subsets and their responses to PS-MPs plus HFD treatment

Through unsupervised clustering analysis of 579 mural cells, we identified four distinct clusters, denoted as MuralCells_1, MuralCells_2, MuralCells_3, and MuralCells_4 (Additional file 1: Fig. S1A). Upon PS-MPs treatment, the proportion of MuralCells_3 decreased, while the proportion of MuralCells_4 increased in both ND and HFD groups (Additional file 1: Fig. S1B and Additional file 1; Table S2). To elucidate the molecular differences of these mural cell subsets, we visually presented the top DEGs for each subset (Additional file 1: Fig. S1C, D). Furthermore, we performed an enrichment analysis to explore the functional characteristics of MuralCells_3 and MuralCells_4. Remarkably, PS-MPs treatment in the ND group resulted in an up-regulation of oxidative phosphorylation, neurodegenerative diseases pathway, and chemical carcinogenesis-reactive oxygen species pathway in MuralCells_3. At the same time, MuralCells_4 exhibited activation of pathways related to cell migration, regulation of chemotaxis, and enzyme inhibitor activity (Additional file 1: Fig. S1E). Pseudotime analysis was employed to discern the differentiation trajectory of mural cells. The trajectory was observed to initiate from MuralCells_3, and PS-MPs plus HFD treatment induced the differentiation of other cell types (Additional file 1: Fig. S1F). Additionally, the top 8 genes exhibiting differential expression with pseudotime progression were depicted in Additional file 1: Fig. S1G.

### Unraveling the heterogeneity and dynamic responses of mesangial cells to PS-MPs plus HFD treatment

Sub-clustering of 896 mesangial cells using unsupervised clustering analysis revealed four distinct clusters, including MesangialCells_1, MesangialCells_2, MesangialCells_3, and MesangialCells_4 (Additional file 1: Fig. S2A). Subsequent investigation revealed that PS-MPs treatment led to an increased proportion of MesangialCells_1, MesangialCells_2, and MesangialCells_4 while decreasing the proportion of MesangialCells_3 in the ND group (Additional file 1: Fig. S2B and Additional file 1; Table S2). The top DEGs among mesangial cell subsets were visually displayed in Additional file 1: Fig. S2C, D. Additionally, we performed functional enrichment analysis to explore the functional characteristics of MesangialCells_1 and MesangialCells_2. Remarkably, following PS-MPs treatment in the ND group, MesangialCells_1 showed up-regulated various protein-binding-related pathways, whereas MesangialCells_2 exhibited activation of pathways associated with oxidative phosphorylation, neurodegenerative diseases, and thermogenesis (Additional file 1: Fig. S2E). We further employed pseudotime analysis to discern the differentiation trajectory of mesangial cells. The trajectory originated from MesangialCells_1 and PS-MPs plus HFD treatment regressed the differentiation of other cell types (Additional file 1: Fig. S2F). Additionally, the top 8 genes exhibiting differential expression with pseudotime progression were displayed in Additional file 1: Fig. S2G.

## Discussion

Recent evidence indicates that humans constantly inhale and ingest MPs (plastic particles less than 5 mm in size), which raises increasing concerns about their implications for human health [48]. The physicochemical properties of MPs (e.g., shape, size, concentrations, surface charge, and hydrophobicity) affect the transformation, interaction, fate and bioavailability to organisms [49]. The 1 µm-sized PS-MPs have been used in many high-quality studies of the effects of PS-MPs on human health [30, 34, 35]. Furthermore, the presented study provides a comprehensive exploration of the impact of PS-MPs and HFD on the kidney microenvironment using scRNA-seq. The results demonstrate significant changes in cell composition, differentiation trajectories, and functional characteristics of the kidney microenvironment following PS-MPs and HFD exposure. These findings contribute to understanding the complex interactions between microplastics, diet, and the renal microenvironment.

The study's results indicate that combining PS-MPs and HFD exacerbates kidney damage, including glomerular and tubular damage and tubulointerstitial fibrosis. These observations are consistent with previous research highlighting the potential renal toxicity of PS-MPs. Chen et al. first demonstrated that exposure to realistic environmental concentrations of PS-MPs could cause oxidative nephrotoxicity through antioxidant inhibition, impair kidney barrier integrity, and increase the risk of acute kidney injury (AKI) [17]. Meng et al. reported that the kidney weight of the mice decreased and the level of BUN and CRE increased significantly after PS-MPs treatment. Besides, the histological observations exhibited slight tubular damage and varying degrees of inflammatory cell infiltration after PS-MPs exposure [18]. Xiong et al. conducted transcriptome analysis and reported that PS-MPs can induce renal dysfunction and histological changes by promoting oxidative stress, inflammation, renal fibrosis, and eventually kidney damage [19]. Our study first used scRNA-seq to elaborate the harmful effects of PS-MPs exposure on kidney health issue, particularly when combined with an unhealthy diet. These findings underscore the importance of regulating plastic pollution and promoting healthier dietary choices to mitigate potential health risks.

The scRNA-seq analysis revealed significant alterations in epithelial cells’ composition and differentiation trajectories following PS-MPs and HFD exposure. Notably, the differentiation trajectory of renal epithelial cells appeared to be regressed after PS-MPs plus HFD treatment, potentially inhibiting their normal development. The activation of kidney development-related pathways suggests that the treatment may disrupt the natural maturation process of renal epithelial cells. These insights could inform future research on renal development and highlight potential mechanisms underlying the observed kidney damage.

Besides, the study provides valuable insights into the immune cells’ responses to PS-MPs and HFD exposure. The altered proportions of T and B cells, as well as changes in polarization scores of macrophages, indicate significant immune dysregulation within the kidney microenvironment following PS-MPs and HFD exposure. The activation of pro-inflammatory and oxidative stress-related pathways in specific immune cell subsets suggests a potential link between microplastic exposure, diet, and immune dysfunction. Understanding these immune responses is crucial for comprehending the broader implications of microplastic exposure on overall health [32, 50].

Finally, this research employs scRNA-seq technology to identify a PF4^+^ macrophage subset and provide insights into its potential role in promoting renal fibrosis after PS-MPs plus HFD exposure, in accordance with a previous study linking PF4^+^ macrophages to renal fibrosis [47]. The results of intercellular interactions between macrophages and fibroblasts suggest a direct involvement of PF4^+^ macrophages in renal fibrosis. These findings highlight a possible mechanism through which PS-MPs plus HFD treatment may contribute to adverse renal outcomes. Understanding the crosstalk between macrophages and other cell types, particularly fibroblasts, expands our knowledge of renal fibrotic processes and could guide the development of targeted interventions [51].

While the study provides valuable insights, it is essential to acknowledge its limitations. The experimental design and results are based on animal models, and translating these findings to humans requires careful consideration. Additionally, the study primarily focuses on cellular responses at the transcriptomic level; further investigations, such as proteomic and metabolomic analyses, are necessary to understand the underlying mechanisms fully. An important matter to address pertains to the distribution and fate of PS-MPs within mice, an aspect which will be subject to further exploration in subsequent research endeavors. Furthermore, forthcoming investigations should encompass an examination of the long-term effects of PS-MPs and HFD exposure, and potential interventions to mitigate the observed adverse outcomes.

In conclusion, the presented study significantly advances our understanding of the intricate interactions between microplastics, diet, and the renal microenvironment. The findings underscore the need for comprehensive strategies to address plastic pollution and promote healthier lifestyles. The study’s emphasis on immune responses, renal fibrosis, and cellular interactions also provides a foundation for further research in toxicology, environmental health, and clinical medicine.

## Conclusion

In summary, this study first elucidates PS-MPs’ effects in conjunction with HFD treatment on the kidney by scRNA-seq approach. The findings unveil that the combined intervention of PS-MPs and HFD aggravated kidney damage and fibrosis and profoundly reshaped cellular compositions in mouse kidneys. This contributes significantly to understanding the intricate interactions between microplastic pollution, dietary factors, and kidney health.

### Supplementary Information

**Additional file 1: Figure S1.** Mural cells heterogeneity and responses to PS-MPs plus HFD Treatment. (A) UMAP of mural cells colored by four treatment groups (left) and macrophage subsets (right). (B) The proportion of mural cell subsets in the four treatment groups. (C) Heatmap of the top 10 DEGs among mural cell subsets according to log fold changes. (D) Violin plot of the top 3 DEGs among mural cell subsets according to log fold changes. (E) KEGG enrichment analysis of upregulation pathways of MuralCells_3 in PS-MPs group vs. ND group (up), and GO enrichment analysis of upregulation pathways of MuralCells_4 in PS-MPs group vs. ND group (down). (F) Pseudotime trajectory analysis colored by mural cell subsets at varying pseudotime stages (left) and by individual mural cell subsets (right). (G) The top 8 differential expression genes with pseudotime progression among mural cell subsets. **Figure S2.** Unveiling mesangial cells diversity and functional responses to PS-MPs plus HFD. (A) UMAP of mesangial cells colored by four treatment groups (left) and macrophage subsets (right). (B) The proportion of mesangial cell subsets in the four treatment groups. (C) Heatmap of the top 10 DEGs among mesangial cell subsets according to log fold changes. (D) Violin plot of the top 3 DEGs among mesangial cell subsets according to log fold changes. (E) GO enrichment analysis of upregulation pathways of MesangialCells_1 in PS-MPs group vs. ND group (up), and KEGG enrichment analysis of upregulation pathways of MesangialCells_2 in PS-MPs group vs. ND group (down). (F) Pseudotime trajectory analysis colored by mesangial cell subsets at varying pseudotime stages (left) and by individual mesangial cell subsets (right). (G) The top 8 differential expression genes with pseudotime progression among mesangial cell subsets. **Table S1.** The histological activity scores of HE and Sirius Red staining for kidneys of mice. **Table S2.** The proportions of distinct cell subpopulations in the four groups. **Table S3.** The IHC score of α-SMA in PF4+ macrophages.

## Data Availability

The original data are included in the article and supplementary materials. Further inquiries can be directed to the corresponding authors. The authors will offer the raw data supporting the conclusions of this article without undue reservation.

## Abbreviations

AECs: Arterial endothelial cells
CapECs: Capillary endothelial cells
ccRCC: Clear cell renal cell carcinoma
CD8Teff: CD8^+^ effector T cells
cDC1: Conventional type 1 dendritic cells
cDC2: Conventional type 2 dendritic cells
DCT: Distal convoluted tubule
DEGs: Differentially expressed genes
ECM: Extracellular matrix
FUSCC: Fudan University Shanghai Cancer Center
GlomerularECs: Glomerular endothelial cells
GlomerularEpi: Glomerular epithelial cells
HelperT: T helper cells
HFD: High-fat diet
HE: Hematoxylin and eosin
ICs: Collecting duct intercalated cells
IHC: Immunohistochemistry
LOH: Kidney loop of Henle epithelial cells
mIHC: Multiplex immunohistochemistry
ND: Normal diet
NK: Natural killer cells
PCs: Collecting duct principal cells
PelvisUrothelial: Kidney pelvis urothelial cells
PlasmaCells: Plasma cells
ProliferatingMPs: Proliferating mononuclear phagocytes
ProliferatingT: Proliferating T cells
PS: Polystyrene
PS-MPs: Polystyrene microplastics
PT: Proximal tubule cells
ROS: Reactive oxygen species
scRNA-seq: Single-cell RNA sequencing
UMAP: Uniform manifold approximation, and projection
