# Supplementary figures and images for: Single-cell RNA-seq analysis decodes the kidney microenvironment induced by polystyrene microplastics in mice receiving a high-fat diet
Source: J Nanobiotechnology. 2024 Jan 3;22:13. doi: 10.1186/s12951-023-02266-7 (PMC10762848; doi:10.1186/s12951-023-02266-7)

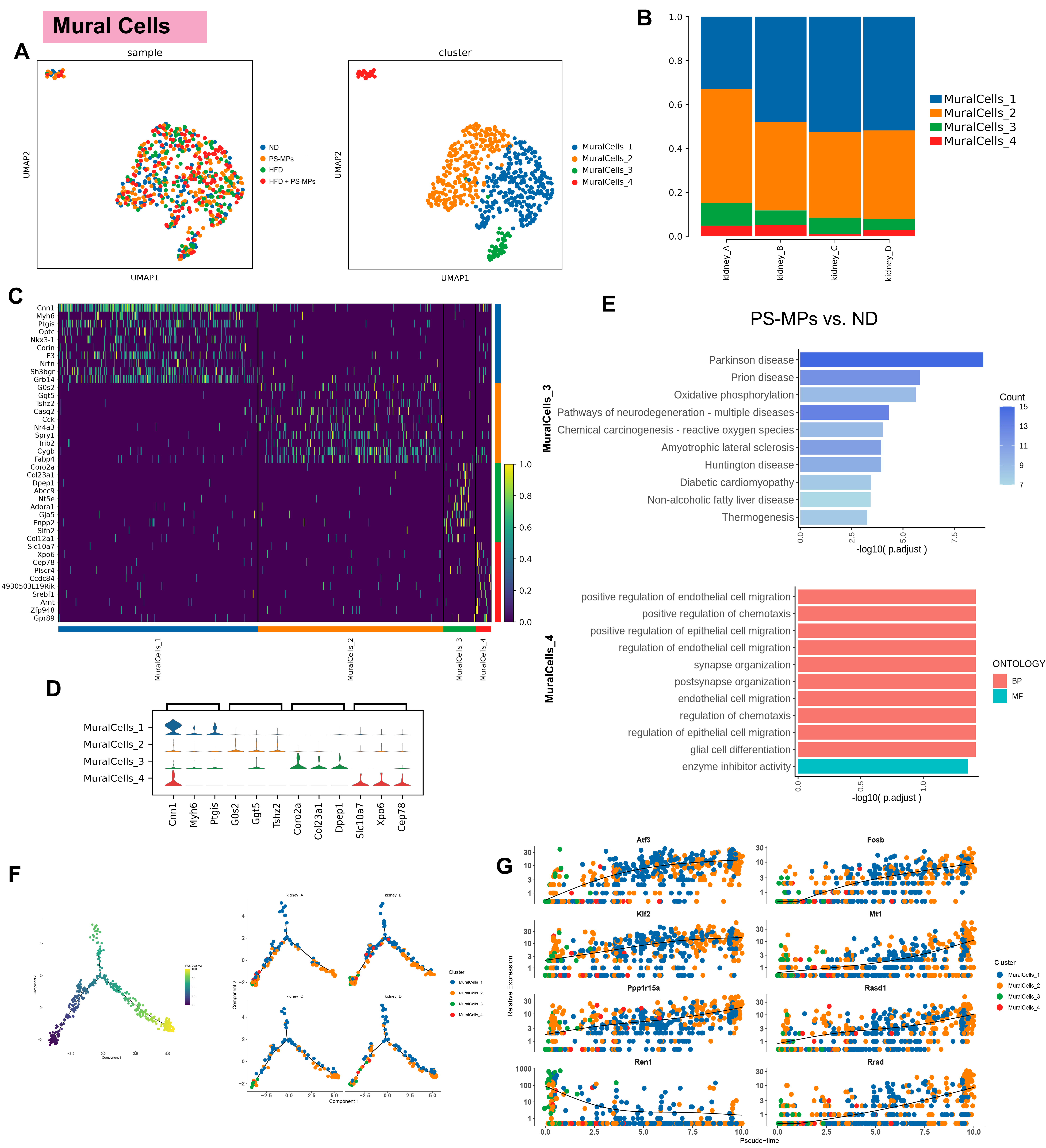

Supplement: Supplementary file 1 — Additional file 1: Figure S1. Mural cells heterogeneity and responses to PS-MPs plus HFD Treatment. (A) UMAP of mural cells colored by four treatment groups (left) and macrophage subsets (right). (B) The proportion of mural cell subsets in the four treatment groups. (C) Heatmap of the top 10 DEGs among mural cell subsets according to log fold changes. (D) Violin plot of the top 3 DEGs among mural cell subsets according to log fold changes. (E) KEGG enrichment analysis of upregulation pathways of MuralCells_3 in PS-MPs group vs. ND group (up), and GO enrichment analysis of upregulation pathways of MuralCells_4 in PS-MPs group vs. ND group (down). (F) Pseudotime trajectory analysis colored by mural cell subsets at varying pseudotime stages (left) and by individual mural cell subsets (right). (G) The top 8 differential expression genes with pseudotime progression among mural cell subsets. Figure S2. Unveiling mesangial cells diversity and functional responses to PS-MPs plus HFD. (A) UMAP of mesangial cells colored by four treatment groups (left) and macrophage subsets (right). (B) The proportion of mesangial cell subsets in the four treatment groups. (C) Heatmap of the top 10 DEGs among mesangial cell subsets according to log fold changes. (D) Violin plot of the top 3 DEGs among mesangial cell subsets according to log fold changes. (E) GO enrichment analysis of upregulation pathways of MesangialCells_1 in PS-MPs group vs. ND group (up), and KEGG enrichment analysis of upregulation pathways of MesangialCells_2 in PS-MPs group vs. ND group (down). (F) Pseudotime trajectory analysis colored by mesangial cell subsets at varying pseudotime stages (left) and by individual mesangial cell subsets (right). (G) The top 8 differential expression genes with pseudotime progression among mesangial cell subsets. Table S1. The histological activity scores of HE and Sirius Red staining for kidneys of mice. Table S2. The proportions of distinct cell subpopulations in the four groups. Ta [file 12951_2023_2266_MOESM1_ESM.zip › 12951_2023_2266_MOESM1_ESM/Figure S1.tif]

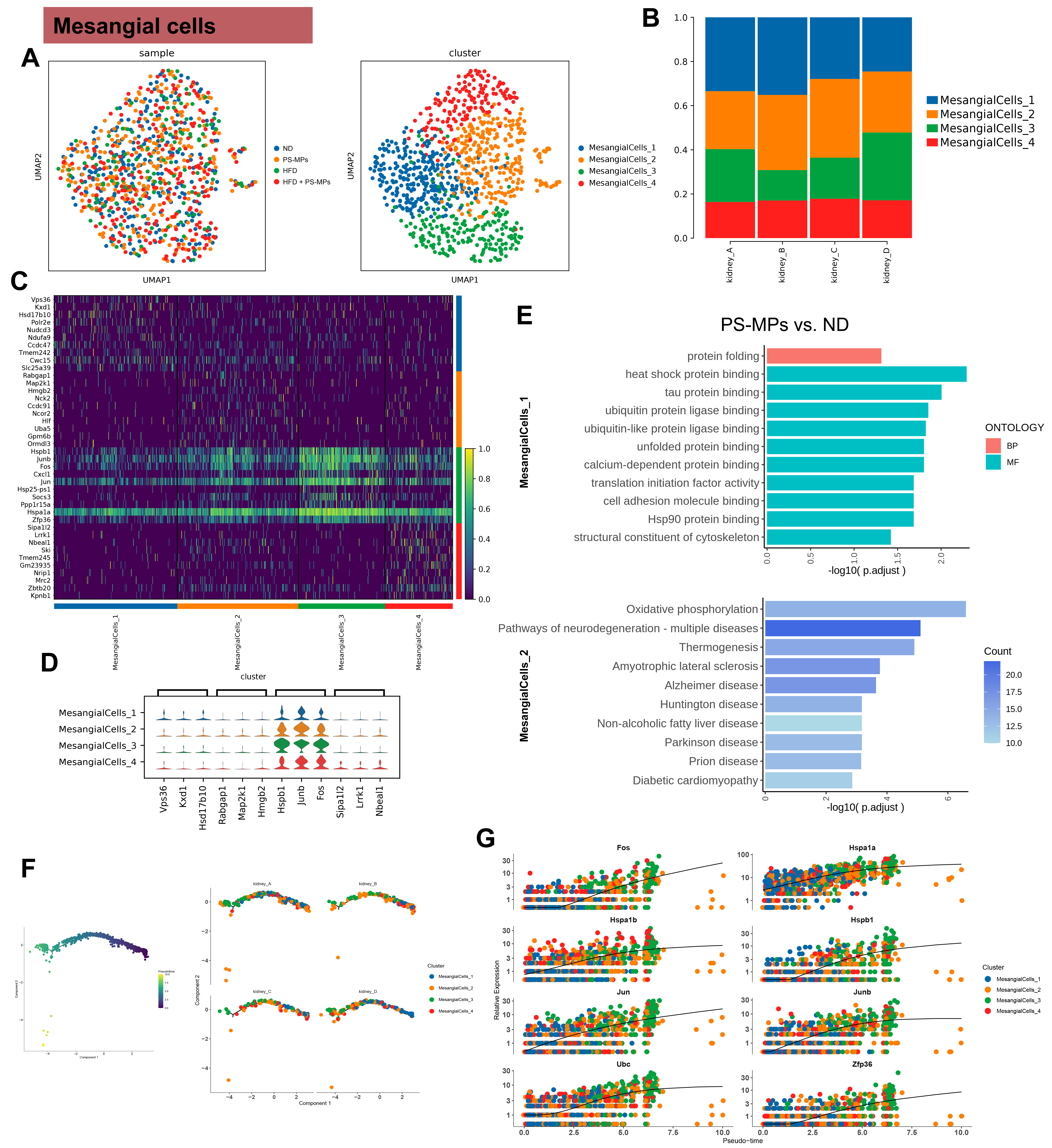

Supplement: Supplementary file 1 — Additional file 1: Figure S1. Mural cells heterogeneity and responses to PS-MPs plus HFD Treatment. (A) UMAP of mural cells colored by four treatment groups (left) and macrophage subsets (right). (B) The proportion of mural cell subsets in the four treatment groups. (C) Heatmap of the top 10 DEGs among mural cell subsets according to log fold changes. (D) Violin plot of the top 3 DEGs among mural cell subsets according to log fold changes. (E) KEGG enrichment analysis of upregulation pathways of MuralCells_3 in PS-MPs group vs. ND group (up), and GO enrichment analysis of upregulation pathways of MuralCells_4 in PS-MPs group vs. ND group (down). (F) Pseudotime trajectory analysis colored by mural cell subsets at varying pseudotime stages (left) and by individual mural cell subsets (right). (G) The top 8 differential expression genes with pseudotime progression among mural cell subsets. Figure S2. Unveiling mesangial cells diversity and functional responses to PS-MPs plus HFD. (A) UMAP of mesangial cells colored by four treatment groups (left) and macrophage subsets (right). (B) The proportion of mesangial cell subsets in the four treatment groups. (C) Heatmap of the top 10 DEGs among mesangial cell subsets according to log fold changes. (D) Violin plot of the top 3 DEGs among mesangial cell subsets according to log fold changes. (E) GO enrichment analysis of upregulation pathways of MesangialCells_1 in PS-MPs group vs. ND group (up), and KEGG enrichment analysis of upregulation pathways of MesangialCells_2 in PS-MPs group vs. ND group (down). (F) Pseudotime trajectory analysis colored by mesangial cell subsets at varying pseudotime stages (left) and by individual mesangial cell subsets (right). (G) The top 8 differential expression genes with pseudotime progression among mesangial cell subsets. Table S1. The histological activity scores of HE and Sirius Red staining for kidneys of mice. Table S2. The proportions of distinct cell subpopulations in the four groups. Ta [file 12951_2023_2266_MOESM1_ESM.zip › 12951_2023_2266_MOESM1_ESM/Figure S2.tif]
